# Women’s experiences of trauma, the psychosocial impact and health service needs during the perinatal period

**DOI:** 10.1186/s12884-023-05509-5

**Published:** 2023-03-21

**Authors:** Essence Perera, Sharon Chou, Nicole Cousins, Natalie Mota, Kristin Reynolds

**Affiliations:** 1 Department of Psychology, University of Manitoba, P313 Duff Roblin Building, 190 Dysart Rd, Winnipeg, MB R3T 2N2 Canada; 2 Department of Psychiatry, University of Manitoba, Winnipeg, MB Canada; 3 Department of Clinical Health Psychology, University of Manitoba, Winnipeg, MB Canada

**Keywords:** Perinatal period, Trauma, Psychological symptoms, Medical care, Health service needs, Psychological health, Qualitative research

## Abstract

**Background:**

Traumatic events are associated with psychological and physical health problems for women in the perinatal period (i.e., pregnancy-12-months after childbirth). Despite the negative impact of trauma on perinatal women, the long-term impact of such diverse trauma and women’s experience during the perinatal period remains understudied.

**Methods:**

This study explored two research questions: 1) What are the psychological experiences of perinatal women who have experienced interpersonal traumatic events? And 2) What are the service needs and gaps expressed by women relating to perinatal medical protocols and psychological services? These questions were addressed via in-depth semi-structured qualitative interviews with nine perinatal women (one pregnant and eight postpartum) residing in central Canada who reported experiencing interpersonal traumatic events occurring from adolescence to the perinatal period. Recruitment and data collection occurred from October 2020 to June 2021. Interviews were audio-recorded, transcribed, and analyzed according to constructivist grounded theory.

**Results:**

The emergent grounded theory model revealed the central theme of the role of prior trauma in shaping women’s perinatal experiences, with four related main themes including perinatal experiences during the COVID-19 pandemic, the role of social support in women’s perinatal experiences, the barriers that women experienced while seeking psychological and medical services prior to the perinatal period and during the perinatal period, and the specific needs of perinatal women with a history of interpersonal trauma.

**Conclusions:**

Findings of this research highlight the negative and long-lasting impact of traumatic events experienced on women’s psychological health and psychosocial functioning during the perinatal period, as well as perinatal women’s unmet psychological and medical service needs. A call to action for perinatal researchers and clinicians is imperative in furthering this important area of research and practicing person-centered and trauma-informed care with this population.

**Supplementary Information:**

The online version contains supplementary material available at 10.1186/s12884-023-05509-5.

## Background

The importance of women’s psychological health while pregnant and after childbirth is well-documented and recognized globally [1]. The perinatal period, extending from pregnancy to 12-months after childbirth [2], is a time marked by several significant physiological, social, and psychological changes, which can negatively impact the psychological health of perinatal women [3, 4] For some mothers, the perinatal period is a time marked by an increased vulnerability to experience events relating to complications in pregnancy or in labour and delivery such as an unplanned cesarian section [5]. Trauma has been defined as a stressful experience involving a serious incident leading to physical harm, loss of life, or violence of a sexual nature that either occurred or was likely to occur [6]. As it pertains to the present study, interpersonal traumatic events can be characterized by a traumatic experience that may have occurred prior to or during the perinatal period and either involved the perinatal woman or an individual they care about [7]. These interpersonal traumatic events involving perinatal women may be related to women’s pregnancy (e.g., pregnancy loss) or unrelated to their pregnancy (e.g., sexual assault), with the potential to lead to significant distress in women [7].

Numerous studies have found associations between traumatic experiences and adverse outcomes for women in the perinatal period, including physical health problems while pregnant such as vaginal bleeding and high blood pressure, psychological problems in pregnancy and postpartum such as anxiety, depression, posttraumatic stress symptoms (PTSS) and posttraumatic stress disorder (PTSD) and complications in childbirth [5, 8–10]. Despite these adverse outcomes associated with trauma, research to date has focussed samples with specific traumatic experiences such as pregnancy loss, intimate partner violence, and traumatic birth [11–13] and not traumatic events more broadly. Further, prior work explored the impact specific to the psychological sequelae following trauma, but less is known about women’s experiences subsequent to trauma. There is a need for understanding during this COVID-19 period in order to impact on interventions involving perinatal women.

In summary, the literature suggests that perinatal women have an increased susceptibility to experience interpersonal traumatic events that lead to psychological problems [5, 8–10]. It is evident that trauma-informed care (an approach utilized by care providers grounded in understanding the needs of those who have experienced traumatic events) is important not only in the context of educating care providers of the various traumatic experiences that women may have and the impacts of such experiences, but also in equipping them with the skills to be sensitive and compassionate [14]. This supports the need for further research to better understand women’s perspectives which will help to inform best practices in perinatal psychological and medical services.

### Perinatal service use

To date, there is limited research that has explored the psychological and medical service use of perinatal women, and even less research that has examined service use in perinatal women who have experienced trauma. According to the literature, perinatal women often find it hard to recognize and distinguish psychological distress from what are ‘normal’ parts of the perinatal period, and thus, are less likely to seek treatment for psychological symptoms [15]. Research by Muzik and colleagues (2013), suggests that a majority of women expressed the importance of receiving care from psychological service providers who considered their needs as women with a history of trauma, these women had experienced maltreatment as children [16]. This research also highlighted that trust in psychological service providers was a key component for perinatal women who reflected on their help-seeking experiences. Qualitative research by Gokhale and colleagues (2020) identified several themes, one of which suggests that discussing the details of traumatic experiences with medical care providers can be potentially beneficial for perinatal women who perceive conversation to be helpful or retriggering for those who do not perceive the disclosure to be helpful [17]. Another emergent theme in this research expressed the importance of developing a connection between the patient and the health professional in any trauma inquiry and support [17].

The extant body of research presented above supports that little is known regarding the psychological and medical service use in perinatal women who have experienced various types of interpersonal traumatic events. Additionally, perinatal women experience barriers in accessing and receiving perinatal psychological services at multiple levels including personal (e.g., stigma and associated shame) and systemic (e.g., insufficient knowledge of, and access to information and resources) [18, 19]. Those experiencing psychological symptoms may not seek help for their symptoms due to the stigma associated with psychological services. This stresses the importance of identifying and addressing barriers to accessing and receiving care in perinatal women. There remains a gap in the literature regarding perinatal women’s unique perspectives on the current psychological and medical services and whether there are ways to improve these services for women with a history of trauma.

## Objectives

The current study recognizes the importance of individual experiences associated with trauma among women and as such, followed a constructivist grounded theory design employing semi-structured in-depth individual interviews to address two main objectives: 1) To explore the psychological symptoms and experiences of perinatal women with histories of interpersonal traumatic experiences (prior to, or during pregnancy); and 2) To understand the psychological and medical service needs and gaps as expressed by perinatal women.

## Method

### Research strategy

The current study used a mixed-methods strategy, predominantly qualitative in design. In order to address the main objectives of this study, a constructivist grounded theory design was utilized [20]. With the use of constructivist grounded theory, this allowed for the novel opportunity to create a richer understanding of the experiences of perinatal trauma as this approach allows for the analysis remain close to the data. This approach informed data collection and analysis, as in this methodology, data collection and analysis occur simultaneously (further details are discussed later in Analytic approach) [20].

### Participants

Women who were: a) at minimum 18 years old, b) were pregnant or postpartum (up to 1 year after childbirth) and, c) self-reported a history of at least one interpersonal traumatic experience in the past and/or that related to their pregnancy, labour and delivery, or postpartum experience were eligible for study inclusion. This study was approved by the Research Ethics Board at the University of Manitoba, and the Research Board at Women’s Health Clinic Birth Centre.

### Recruitment

Participants were recruited from community mental health self-help organizations, community perinatal organizations, and social network groups/sites in Manitoba. A paper copy of the poster was used to recruit participants at all physical locations (of community mental health self-help organizations and community perinatal organizations,) with exception of the Perinatal Mental Health Clinic located in the Psychological Services Centre where the study recruitment advertisement was posted virtually on the Psychological Services Centre website. Participants were recruited through social media platforms where the study recruitment poster was displayed virtually in various public and private groups on Facebook (e.g., Winnipeg Mom’s Group) and organizations (e.g., With Women Doula Collective).

Participants received a $10 Amazon e-gift card as honorarium for participating in this research. Recruitment began in October 2020 and the last interview was scheduled on June 18, 2021. Decisions concerning when to stop recruiting participants were determined based on theoretical sufficiency, in line with the constructivist grounded theory method [21]. In line with grounded theory data collection and analysis were conducted in parallel, thematic categories were sufficiently explained upon reaching interview 9.

### Data collection procedure

Participants completed telephone screening prior to consent and study enrollment to ensure that they met eligibility criteria. To ensure eligibility, participants were asked the following screening questions: “Have you ever experienced an event in your life where you have feared for your life or for your physical safety? Have you ever experienced an event in your life where you have feared for the life or the physical safety of someone you love? Have you ever witnessed someone else being seriously injured or involved in sexual violence?” If a participant answered yes to any of the above screening questions, they were sent the consent and questionnaire package. After their age was determined to be at a minimum of 18 years, they were then scheduled to participate in a virtual qualitative interview. Participants completed an online consent form and questionnaire package through Qualtrics (Qualtrics, Provo, UT) followed by a semi-structured in-depth qualitative virtual interview approximately one week later. The questionnaire package included standardized measures to assess anxiety, depression, and PTSD as well as a background questionnaire (Edinburgh Perinatal/Postnatal Depression Scale, Perinatal Anxiety Screening Scale, and The Posttraumatic Stress Disorder Checklist for the DSM-5 with Life Events Checklist) [22–24]. The background questionnaire asked about participants’ sociodemographic information (e.g., age, education), perinatal status and experiences (e.g., whether they were currently or previously pregnant, if they have experienced neonatal loss), self-reported physical and psychological health (e.g., general physical health, general psychological health), and psychological help-seeking history (e.g., whether they sought help from a professional for their anxiety, depression, or PTSD).

### Qualitative interview

The virtual qualitative interviews were conducted through Zoom Professional, were facilitated by the same trained interviewer across all interviews and were approximately 60–90 min in length. Interviews were audio-recorded, transcribed verbatim with Trint automated transcription software (Trint Limited), and copy-edited to ensure accuracy. The interview protocol contained questions about the ways in which participants’ trauma and/or psychological symptoms impacted their experiences during the perinatal period, access to psychological health services, and ways in which their experiences could have been improved through revisions to medical care/ protocols or access to services.

### Ethical considerations and actions

There were several ethical considerations taken by the authors as it relates to this study and the sensitivity of trauma research. Prior to the phone screening, participants were informed via email that they would be asked a few questions about a stressful/distressing experience. Participants were given the information that they could respond with a dichotomous (yes or no) response to the questions and no details were required. Additionally, at the time of the phone screening, participants were informed that if they were feeling distressed, they could stop the phone screening questions at any time or refuse to answer. When participants were asked to complete the quantitative measures through Qualtrics, recognizing that asking participants questions about their psychological symptoms and stressful experiences may be distressing, the authors provided local psychological services and crisis resources to the participants. At the scheduled virtual qualitative interview, prior to asking the semi-structured questions, the interviewers discussed with participants that they can stop or take a break during the interview at any time. The interviewers also asked participants about their psychological health state and how they were feeling at the end of the interview. Participants were provided with the contact information for local psychological services and resources at the end of the interview.

### Quantitative measures

#### Sociodemographic variables

Data regarding participants’ age, sex, perinatal status (i.e., pregnant, or postpartum), marital status, ethnicity, highest level of educational attainment were collected.

#### *Edinburgh Perinatal/Postnatal Depression Scale (EPDS) *[22]

The EPDS is a 10-item self-report measure, that was used to assess depression symptoms experienced in the perinatal period [22]. The authors excluded item 10 for ethical consideration purposes, which assessed the frequency of self-harm thoughts with the following question: “The thought of harming myself has occurred to me.” As such, total scores represent responses out of nine items. Participants used a 4-point Likert scale to respond to the frequency of experiencing depressive symptoms over the past seven days with scale anchors that differ based on the question asked (e.g., 0 = As much as I always could or 0 = No, not at all; to 3 = Not at all or 3 = Yes, very often). Total scores of 13 or higher indicate a high risk of perinatal depression [22]. Previous research indicates that this measure has excellent internal reliability (Cronbach’s α = 0.87) [22].

#### *Perinatal Anxiety Screening Scale (PASS) *[23]

The PASS is a 31-item self-report measure that was used to assess perinatal anxiety symptoms [23]. Respondents used a 4-point Likert scale (from 1 = Not at all to 4 = Almost always) to indicate the frequency of anxiety-related symptoms they experienced in the past month. Total scores of 26 or higher indicate a high risk of perinatal anxiety [23]. This measure was found to have excellent internal reliability (Cronbach’s α = 0.96) [23].

#### *The Posttraumatic Stress Disorder Checklist for the DSM-5 (PCL-5) with Life Events Checklist 5 (LEC-5) *[24]

This version of the PCL-5 is comprised of two parts [24]. The first portion is comprised of 17 items in which participants indicated whether each event has directly or indirectly happened to them. The second portion is comprised of 20 items that was used to assess symptoms of PTSD. Participants used a 5-point Likert scale (from 0 = Not at all to 4 = Extremely) to respond to the frequency of PTSD symptoms they experienced in the previous month. A cut-off score between 31–33 indicates that the participant may meet criteria for a PTSD diagnosis, and further assessment is needed [24]. Previous research suggests the PCL-5 demonstrates strong internal consistency (α = 0.94), test–retest reliability (*r* = 0.82), and convergent (rs = 0.74 to 0.85) and discriminant validity (rs = 0.31 to 0.60) [25].

### Analytic approach

Qualitative data were analyzed based on the Constructivist Grounded Theory (GT) [20]. Constructivist applies to the emergent theories from the data, that are co-created by the participants and researchers involved [20]. This methodology employed procedures for data collection and analyses that are based on the fixed and flexible foundations of grounded theory [20]. In this methodology, codes emerged from the concurrent data collection and analysis and flexible, interconnected application of line-by-line (initial), focused, and theoretical coding of transcripts that led to the development of a thematic framework [20]. Initial coding involved a two-tiered approach: first the authors engaged in the familiarization phase and reviewed the entire interview transcript to gain a general understanding of the experiences the participant expressed, then the authors coded the data line-by-line, staying close to the data and coding for action-oriented words. For example, when a participant described their perinatal experiences she said, “There's a lot of unknown and with that comes a lot of fear and anxiety of trying to be the right parent, even though everybody has so many different styles of how-to parent. And just trying to like, there's so much new information out there that how do you know what's the right thing to do?” this was coded as describing perinatal experience and the way it was described as ‘fear and anxiety of trying to be the right parent’. And then in focused coding, codes became larger in scope, collapsing smaller units of meaning identified in the line-by-line coding phase into larger meaning concepts. Following with the example stated earlier, the initial code of ‘fear and anxiety of trying to be the right parent’ led to the focused code of ‘underlying pressures as a parent’. Theoretical coding involved connecting codes which led to the development of the emergent thematic framework. In this last phase of coding, all data coded were arranged into a coherent narrative. In following with the example, the code ‘underlying pressures as a parent’ was later revised once new data were collected and analyzed to reflect ‘pressure to make right decisions for pregnancy and for baby’. A critical component of this methodology is constant comparison, as new data were collected and compared to data that were previously coded [20]. The authors engaged in memo writing and writing field notes throughout data collection which documented the authors early analytic thinking, in line with the methodology [20].

Quantitative data were analyzed descriptively to provide context to our sample of perinatal women. Quantitative data was examined using descriptive statistics in the form of a summary to indicate the age range of the sample, ethnicity breakdown of the sample, level of educational attainment, perinatal status and marital status of the sample. Data obtained from the EPDS, PASS, and PCL-5 were scored by adding up the participant responses using the Likert-scale anchors [22–24].

### Rigour

To ensure rigour in the present research, several procedures were followed. Since each researcher carries numerous lenses and worldviews with them, the qualitative analysts (EP, SC, KR) met at each step of the analysis (e.g., coding, developing emerging framework) to explore areas of convergence and divergence, and we came to a consensus. After each qualitative interview, EP recorded field notes which included interview observations and impressions concerning the processes and dynamics between the interviewer and participant. Thick description [26] of women’s experiences and impact of trauma in the perinatal period was achieved through in-depth virtual qualitative interviews, memo writing (documentation of emergent analytic thoughts), writing observational field notes, and keeping a reflexivity journal. The purpose of the reflexivity journal is related to a few different factors: a) it helped the first author and lead analyst to be aware of their own biases throughout the interview, analyses, and write-up of the manuscript; and b) it helped the lead analyst to note their interpretations without confounding personal views with participants’ experiences. Credibility was achieved through confirmation of multivocality, where careful attention is paid to the multiple perspectives shared by the participants in the interviews [26, 27]. Through these methods, the emergent thematic framework remained close to the data collected and thus is aligned with the voiced expressed by the participants.

## Results

### Sample characteristics

All women interested in participating were screened into the study by EP (total of ten women). One participant did not attend the virtual qualitative interview and was not responsive to reschedule. As such, this participant’s quantitative data were excluded from analysis. Nine perinatal women between the ages of 25 and 39 were included in this study. One participant was pregnant, and the remaining participants were postpartum (one to twelve-months after childbirth). There were consistencies in experiences among the one pregnant woman and other postpartum women. Also, important to address that even though there was one pregnant participant at the time of data collection, all participants had the experience of being pregnant within the past year and shared experiences related to their pregnancies throughout interviews. A majority of participants identified as White, one participant reported Indigenous background (First Nations, Metis, Inuit) and most had a university undergraduate degree (77.8%). All participants were married or common-law. Three women had experienced a neonatal loss. Most of the sample self-reported an experience of anxiety, depression, or PTSD in their lifetime (7/9), and five of which reported that they sought help for these experiences. Data collected from the EPDS, PASS, and PCL-5 are summarized in Table 1 [22–24]. Five participants met the threshold for probable depression based on the EPDS, four participants met the threshold for severe symptoms of anxiety according to the PASS, and three participants met the clinical cut-point for PTSD based on the PCL-5 [22–24]. Participant 3 discussed three traumatic events whereas all other participants each spoke about single traumatic events. Perinatal women discussed varied and distinct traumatic events including loss (loved one, colleague, or fetal loss); serious illness or accident; interpersonal (infidelity, divorce, sexual abuse); and labour and delivery (unplanned cesarian section, and complications with labour and delivery). Despite these varied trauma types, the important role that these events had on shaping women’s experiences during the perinatal period was ubiquitous.

**Table 1:** Psychological symptoms of perinatal women

Domain	Mean Score	Range of Total Scores	Number of Women Who Met Clinical Cut-Points for Diagnosis	Clinical Range/Screen Interpretation
EPDS^a^	13.9	6–19	5	13 + Probable depression*
PASS	34.1	4–64	4	42 – 93 Severe symptoms of anxiety*
PCL-5	24.1	3–55	3	31–33 Clinical cut-point score for PTSD*

Sample data collected from three standard measures are presented in this table. EPDS—Edinburgh Perinatal/Postnatal Depression Scale, PASS—Perinatal Anxiety Screening Scale, PCL – 5 The Posttraumatic Stress Disorder Checklist for the DSM-5 [22–24]. Clinical cut-points are indicated with an asterisk[*] next to the clinical range/screen interpretation

^a^EPDS mean and total scores are based on 9 items and therefore it is possible that some of the participants could have had an even higher score [22]

### Qualitative findings

**Fig. 1:**
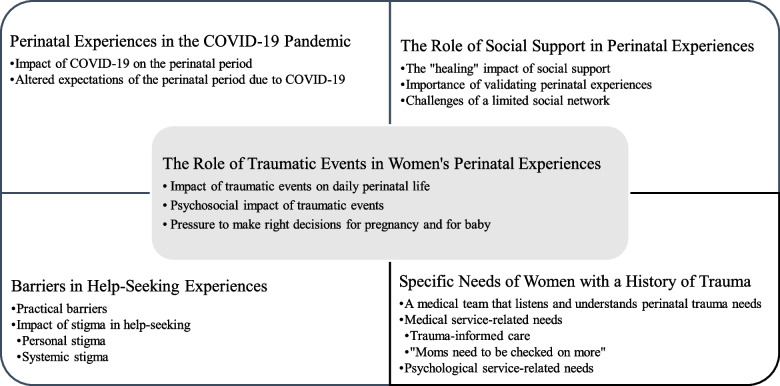
Grounded theory model of perinatal women’s experiences of trauma, psychosocial impact and medical and psychological service needs. Emergent themes from qualitative analysis of semi-structured interviews with perinatal women

In-depth semi-structured qualitative interviews with nine perinatal women highlighted several important themes (see Fig. 1). The core thematic category emergent from the analysis highlighted the important role of prior histories of trauma on women’s perinatal experiences. Subthemes within this main theme included: a) the impact of traumatic events on daily perinatal life, b) psychological impact of traumatic events on perinatal women, and c) pressure to make the right decisions for pregnancy and for baby. The second main theme illustrated perinatal experiences during the COVID-19 pandemic, with subthemes highlighting: a) the impact of the COVID-19 pandemic on perinatal women with trauma histories; and b) the ways in which expectations of the perinatal period were altered due to the COVID-19 pandemic. The third main theme illuminated the role of social support in women’s perinatal experiences, with subthemes supporting: a) the “healing” impact of social support; b) the importance of validating perinatal experiences; and c) the challenges of a limited social network. The fourth main theme demonstrated the various barriers that women experienced while seeking psychological and medical services prior to and during the perinatal period with subthemes describing: a) practical barriers; and b) the impact of stigma on help-seeking including personal and systemic stigma. The fifth and final main theme exemplified the specific needs of women with a history of interpersonal trauma, with the following subthemes: a) a medical care team that listens and understands perinatal trauma needs; b) medical service-related needs such as trauma-informed care and “moms need to be checked on more”; and c) psychological service-related needs (see Summary of recommendations made by perinatal women in Table 2). Each of these main and subthemes will be further described with supporting qualitative evidence in the sections that follow.

**Table 2:** Summary of recommendations made by perinatal women

Recommendation #1: A humanized care model is important, in a medical system that de-humanizes. Perinatal women recommended that health providers call women by their names. Also, medical staff that is helpful, responsive, attentive, and supportive
Recommendation #2: Perinatal women recommended that care providers acknowledge and validate their experiences. It is imperative that medical providers apply the appropriate emotions in circumstances that may be difficult for the patients
Recommendation #3: Perinatal women indicate that partners should be included in appointments and adequately informed of the experiences and changes that occur in the perinatal period. Partners should be taught to recognize and watch out for psychological and physical symptoms that must be addressed with health professionals
Recommendation #4: It is important that medical professional recognize and acknowledge that certain medical examinations may be distressing to women who experienced trauma. It is imperative that medical policies consider the need for a support person to accompany the patient while an uncomfortable examination is taking place
Recommendation #5: There is a need for trauma-informed medical care that respects women’s rights. Women expressed that medical care providers must indicate initial point of contact by verbally communicating that the exam will begin followed by physical touch to the knee prior to performing the exam can be helpful when working with patients who have experienced trauma. With regards to improvements that can be made to facilitate a positive experience while seeking medical services, perinatal women spoke about the importance of a trauma-informed approach to medical care. It is also recommended that awareness about trauma is incorporated into learning
Recommendation #6: Access to private rooms was a central topic discussed by many women as in the case of pregnancy loss or complications in childbirth. It is important to consider the specific needs of women who have experienced a traumatic event to not further distress these women
Recommendation #7: As explained by a perinatal woman “Moms Need to be Checked on More.” There is a need for more support for those who experienced pregnancy or neonatal loss. Follow-up care is needed from health professionals for women who experience pregnancy loss as there are many psychological and physical issues that may accompany this type of loss (e.g., “overwhelming sadness”, breast milk may come in days after a late-term pregnancy loss). Additionally, there is a need for support programs and services that address issues perinatal women may experience such as with breast-feeding. Further, health professionals should listen to the issues perinatal women are experiencing and work with them to come up with appropriate next steps (e.g., referrals to specialists)
Recommendation #8: Perinatal women suggest that improvements can be made in accessing psychological services including increasing the availability of counselling services that are easily accessible, particularly, easier access in rural areas while also reducing the cost and a ‘what to expect’ guide for important areas to cover during your first psychological appointment
Recommendation #9: For women expressing distressing psychological or emotional issues, the use of a checklist may be helpful as opposed to an open-ended detailed questionnaire when determining if further attention is needed from a psychological professional. Additionally, it is critical that medical providers consider all aspects of health with their patients. Psychological health is an important component of health that should be addressed

### The role of trauma in women’s perinatal experiences.

The central role that traumatic events played in shaping women’s experiences during the perinatal period emerged across interviews. The two subthemes within this main theme include the impact of traumatic events on daily perinatal life and pressure to make the right decisions for pregnancy and for baby. Women described various interpersonal traumatic events occurring from adolescence to the perinatal period. Regardless of the type of traumatic event experienced, perinatal women described commonalities in the psychological sequelae and negative psychosocial impact of trauma in affecting their daily life.

#### Impact of traumatic events on daily perinatal life

Interviews with women illustrated the impact of their traumas on their daily lives and perspectives in the perinatal period. They perceived their traumatic events as impacting them for months to years following the traumatic event, leading to experiencing increased anxiety in the perinatal period. Examples of this increased anxiety voiced by participants included excessive checking for fetal movements while pregnant and checking if the baby is breathing as the baby is sleeping when postpartum. For women who experienced labour and delivery trauma, situations that served as reminders of the traumatic event led to the re-experiencing of distressing emotions. For example, one perinatal woman shared that when she was pregnant, she was often concerned about whether her fetus was alive and reflect on this worry as relating to a time of loss where her mother had a miscarriage. She then expressed how her mother’s miscarriage had impacted her at the time and was possibly the reason why she had been worried about her fetus during her pregnancy. Another woman spoke about her experience in a challenging labour which involved an unexpected cesarian-section that affected her for several months postpartum. She explained that she actively avoids cues and situations that reminded her of the traumatic event she experienced. She described that when television shows portray events like childbirth which conflict with her experience, as she was unable to hold her child immediately after the infant was delivered, and she now avoids any triggering cues related to labour in postpartum. She expressed:

Even right now, it's seeing scenes of childbirth, like in movies and TV shows, I will do something else when that is playing on the TV, I will completely distract myself and do the dishes and play with my [baby] and do a whole bunch of stuff so I'm not paying attention to that scene. Because I want to cry or scream or just shout, why didn't I get that, I didn't get that at all. (Participant #3)

Another perinatal woman experienced medical complications in labour and delivery where there was a risk of death to herself and her fetus, which led to an extended hospital stay while postpartum and included numerous follow-up appointments postpartum for them both. She explained how the traumatic event changed her overall perspective on life and death, where she experienced daily worst-case-scenario fears concerning her own death and the death of her baby:

I had never had to... be worried about like living or dying before, I never had that concept before, so now it just makes you not like appreciate things more, but just to be more aware of the seriousness of certain things. (Participant #4)

#### Psychological impact of traumatic events

The psychological impact of traumatic events on perinatal women were such that each of the women expressed similar feelings of being overwhelmed, worried, and experiencing low mood. For example, a perinatal woman who experienced the loss of a loved one prior to the perinatal period shared that the effects of trauma continued into the perinatal period, “I experienced sort of a lot of those overwhelming like, I can't do this. feeling anxious. There's like the weight of the world on my shoulders for the majority of my maternity leave.” (Participant #1). Feelings of helplessness and hopelessness were evident in interviews with women who experienced multiple traumatic events. For example, one perinatal woman experienced a challenging divorce which brought up feelings of helplessness and a sense of losing control, and these same feelings were brought up again following a challenging childbirth:

I felt a lot of, very overwhelming, the fact that I couldn't help her in any way, I felt completely helpless and a lot of that definitely felt very familiar too. I felt very helpless in processing everything that I had to do with my divorce. I couldn't, it was happening, and I felt like I lost complete control of my life. And when my [baby] was born and she wasn't with me, I felt like I again lost complete control of everything. (Participant #3)

One woman who experienced pregnancy loss expressed that in subsequent pregnancies she experienced anxiety and persistent fears of losing her fetus, “We lost three babies before [baby] and the anxiety of just even the fear of losing her was there consistently. And the fear that something was always going to be wrong was always there. And it was just really hard to enjoy being pregnant because it was so, so fearful.” (Participant #6). Additionally, this woman explained how isolating it was to be in her position as there was a lack of resources in her area to manage issues related to infertility and pregnancy loss, merged with the lack of support she felt from family and friends. Another woman who experienced late term pregnancy loss shared that this traumatic experience had led to many heavy emotional and psychological impacts including “overwhelming sadness” for many years (Participant #7). She expressed that she was not able to sleep for several nights after her pregnancy loss, and over the course of several years she was not able to visit her friends who had a baby because she could not bring herself to park in the same parkade or enter the hospital doors. She explained, “[The traumatic event] was in [year]. It probably took me, I would say, when I had my [baby] six months ago [six years later] to kind of recover from that,” (Participant #7).

#### Pressure to make the right decisions for pregnancy and for baby

Anxieties and worries related to parenting emerged as an important theme. For women who have experienced a traumatic event, this led to an increased hypervigilance to safety, an increased need to want to do things 'the right way' in order to avoid bad things from happening. There is a sense of overwhelming feelings and helplessness that may arise from trauma, which is triggered by stressful experiences such as being pregnant and having a baby. Women described their ideals concerning what the “right parent” had meant to them, which included breast-feeding their infants, and protecting infants from harm. The pressure of being the “right parent” gave way to increased worries about their ability to take care of their infants which was experienced as overwhelming. For example, one perinatal woman described:

There's a lot of unknown and with that comes a lot of fear and anxiety of trying to be the right parent, even though everybody has so many different styles of how to parent. And just trying to like, there's so much new information out there that how do you know what's the right thing to do? (Participant #3)

The increased pressure to feed and sustain baby and the overwhelming pressure to breastfeed appeared to take a toll on the psychological health of women. One woman, who experienced the loss of a loved one prior to the perinatal period, expressed the heavy weight of the responsibilities of parenting she experienced, “Her well-being was only fully in my hands because I was literally the only source of food for her.” (Participant #1). A perinatal woman shared how her medical care staff spoke about how important breastfeeding is, but she found that there was little support when she experienced issues with breastfeeding. Additionally, this pressure she felt as the primary parent coupled with a lack of awareness of lip and tongue tie issues on the part of medical professionals, led the perinatal woman to further experience distressing psychological symptoms. She explained:

While also not sleeping because I had a newborn and dealing with my own pain and trying to sort out, like breastfeeding issues and trying to get help with that. Yeah, it was it was very, very hard. (Participant #9)

### Perinatal experiences during the COVID-19 pandemic

The COVID-19 pandemic has led to several challenges with respect to the perinatal period such as changes in routine and increased isolation, as well as a few positive impacts such as more time spent with family. The two subthemes within this main theme include the impact of COVID-19 on the perinatal period and altered expectations of the perinatal period due to COVID-19. Difficulties associated with social isolation including isolation from their social and formal supports, lack of socialization for their babies, and worry about their baby’s development were also echoed in several interviews. The role of the pandemic in the perinatal experience can be described as, one woman expressed, a series of “highs and lows” (Participant #5).

#### Impact of COVID-19 on the perinatal period

Women’s experiences during the perinatal period were coloured by the many changes associated with the COVID-19 pandemic. Unexpected changes appeared to intensify psychological symptoms. The pandemic-related closure of several programs and services that were important to the women culminated in their isolation from informal (e.g., extended family and friends, mom groups) and supports. These programs, such as “Mommy and Me” (Participant #1) groups were important to women as it gave them a place to socialize, connect, and build relationships with other moms, normalizing the many challenges of the pregnancy and postpartum periods. Women also shared their views that the effects of isolation from pandemic-related restrictions appeared to exacerbate prior, and sometimes bring on new, symptoms of anxiety and depressed mood. For example, one perinatal woman, for whom social interactions were an integral part of her overall well-being, saw the exacerbation of her prior symptoms of anxiety and depression as being a direct consequence of the isolation associated with lockdowns:

I think [the COVID-19 pandemic] exacerbated every symptom. Because we're not supposed to just live in our houses and be isolated. And I'm very, very social, and very extroverted. So, we literally went from like when I was off with my [baby], we literally went from being in run time, and a ‘mom and me’ group and going to like a feeding support group and going to the going to swimming lessons. We had something every single day of the week to nothing…And so I found myself talking to the dog and I just felt like just I guess just not having the supports there. (Participant #1)

The importance of maintaining a positive perspective about the pandemic or seeing the silver lining of pandemic-related changes was also a salient theme in women’s interviews. They reflected on the aspects of the pandemic that have had positive impacts on them during the perinatal period. A few women shared a sense of gratitude to be safe, illness-free, and to have more time to spend with their families at home. The importance of remaining positive appeared to facilitate adaptive coping and adjustment to motherhood amidst the various uncontrollable and unpredictable situations that the pandemic had created. This positive outlook enabled some women to reflect over the year as the one in which their child was born and in which they were able to spend much of their time at home watching their child grow. One perinatal woman, who works as a healthcare provider in a high-stress environment, shared that the pace at her workplace slowed during the pandemic, which allowed her to feel more relaxed while at work during pregnancy:

I think the pandemic actually kind of had as crazy it sounds a positive twist because I don't know if you know, but like the hospitals, when the pandemic first hit, people were scared to go. My job went from being so busy and stressful to, like, relaxed I could spend time with my patients. (Participant #2)

#### Altered expectations of the perinatal period due to COVID-19

The pandemic also appeared to shift the expectations that women had of their experiences during the perinatal period. Specifically, some women shared that they were not able to take part in many of the positive experiences that typically accompany the perinatal period such as baby showers and visits from family and friends after childbirth. Emotions such as sadness and guilt accompanied the experience of “missing out” (Participant #4). Some women spoke about feelings of guilt owing to an inability to socialize their children with other children. This guilt was also associated with worries for their children’s learning and development. One participant reflected on her pandemic-related experiences in the perinatal period, where her expectations for her maternity leave did not align with the events that occurred. This led to the experience of guilt associated with this conflict and worries for her child’s social development:

I'm also slightly disappointed because my expectations for my maternity leave are very, very different than what my expectations have been. And then to maybe like a little bit feeling of guilt just for my [baby], the social aspect so, I know it has nothing to do with me necessarily, but just feeling a little guilty that she might be missing out on all these great plans that I had for the first year. (Participant #4)

### The role of social support in women’s perinatal experiences

Social support played a protective role in shaping women’s experiences of psychological symptoms and psychosocial impact during the perinatal period. The three subthemes within this main theme include the “healing” impact of social support, the importance of validating perinatal experiences, and the challenges of having a limited social network. The role of social support emerged as an important aspect of a positive perinatal experience, helping to alleviate psychological symptoms in instrumental ways (e.g., having someone to provide childcare so they could go grocery shopping, give them a break when they did not have much sleep the night before) as well as emotionally (e.g., someone to talk to). Women who were able to rely on social support shared the impact this had on their adjustment to motherhood. Support enabled women to manage their new routine and balance parenting while also taking care of themselves. Social support also influenced women’s perceptions toward seeking psychological services. Women who did not have social support described enhanced challenges in adjusting to and coping with changes in the perinatal period (e.g., having to attend prenatal appointments alone).

#### The “healing” impact of social supports

The social supports in practicalities of day-to-day life helped women maintain a sense of balance in their lives, while emotional supports allowed women to feel as though they had a safe space to discuss challenges of adjusting as a parent. Having a close friend or family member to share in-depth information about their emotional well-being emerged as important in women’s perinatal experiences. One perinatal woman reflected on how important it was for her to be able to share her experiences relating to her psychological and emotional well-being with her partner, and how he shifted the way he talked to her from being dismissive to being more empathetic, “Just being an empathetic, listener, instead of just saying, "oh, no, you're fine, you're fine, cheer up.” (Participant #4). The same woman shared that she was able to manage her psychological symptoms of worry associated with her past trauma because of the support she received from her family, “I think it would be a lot more difficult. I think it stopped any of those [negative] feelings from becoming prolonged. I think it helped me deal with it, not quicker, but just to heal faster. So, I think that was good.”

#### The importance of validating perinatal experiences

The availability of social support played a critical role in helping women to feel less alone in their struggles. The importance of validation was illustrated nicely in one perinatal woman’s interview who spoke about a family member’s encouragement to seek out psychological services by giving a poignant analogy, “a car always needs to be [maintained], right. Like, you got to go for an oil change. So, you're essentially going for your own personal oil change.” (Participant #5). Having this conversation allowed this participant to feel validated in her need for professional support, altered her perspective with regards to therapy, and prompted her to take the next step in seeking psychological services.

One woman described her experience with a faith-based peer support group developed within her community, and how important it was for her to find a group of people who had experienced a similar traumatic event as her:

It was more beneficial than for me going to a counselor because the counselor didn't really know how to feel those feelings or to know what it's like to lose a child or anything like that. Then once I found a group of friends that did, it was more benefit because I could feel like, ok, I know that these are just feelings and I'm allowed to feel them and they're normal because they're feeling them too. So, it just made me feel a lot more normal as a human being to have a group of people that I can talk to. (Participant #6)

#### Challenges of a limited social network

In the absence of social support, women described challenges balancing the various aspects of their daily lives (e.g., childcare and housework) and struggling with emotional issues which led to feelings of emotional and physical exhaustion. These women expressed that they needed more emotional and practical support, but family and friends were either unavailable to help due to pandemic-related reasons (e.g., family lives in another country and could not travel due to restrictions, not able to have anyone outside of the household visiting) or they were unsupportive of their needs (e.g., did not offer to help or allow for the space to talk deeply about emotions). One woman elaborated on her challenging experiences without social support in the perinatal period:

You feel just there's no one there because it's so isolating at times, especially because my husband is only home for two hours before the kids go to bed, so I have them for the majority of the time and my [baby] is only one here, and so she's not really talking so I don't have anyone to talk to all day except for two screaming children that can't tell me what they want. (Participant #6)

### Barriers in help-seeking experiences

Perinatal women shared several barriers they experienced while seeking psychological services including those relating to stigma, mental health literacy, and finances. When women encountered these barriers, it often prevented them from further pursuing professional support and addressing their needs. The two subthemes within this main theme are: practical barriers in help-seeking and the impact of stigma in help-seeking. Within the subtheme of the impact of stigma in help-seeking two further themes emerged, personal and systemic stigma. There were also barriers to receiving medical services, where the knowledge of one perinatal woman’s career led to several invalid assumptions. Further, it was assumed that this woman knew about the labour and delivery process because she worked in the healthcare field. It is important to note that various fields in healthcare have different training programs, and assumptions should not be made based on one’s career.

#### Practical barriers in help-seeking

Women experienced a number of practical barriers in accessing and receiving psychological services. The cost of services and distance travelled from rural areas to receive psychological care, all acted as barriers in women’s their help-seeking experiences prior to the perinatal period. The financial barriers to access psychological services continued to impact women in the perinatal period, also while managing further financial constraints postpartum and providing for their infant on a reduced income in maternity leave. For example, one perinatal woman described her experience while attempting to accesses psychological services to address her symptoms, and the high costs to received psychological services prevented her from addressing her symptoms:

I found that I fell into this gap where I had no benefits while I was on maternity leave and I was on employment insurance, so paying a hundred and fifty dollars for a psychologist each time wasn't something that was realistic for us. (Participant #1)

The changes in restructuring health resources due to the COVID-19 pandemic also acted as a barrier to accessing support from the perspective of perinatal women. For one perinatal woman, the challenges of limited resources in a rural area and the lack of resources available due to restructuring the health system in light of COVID-19, acted as barriers to help-seeking. She explained that “no one's taking new clients, limited hours, limited services,” which left her without access to professional psychological health services (Participant #8). Another barrier that was evident in the case described by one woman who shared her concerns for privacy due to the shift from in-person to virtual methods of therapy in response to the ongoing pandemic-related changes:

Talking about it at home, it's like I don't know if my boyfriend can hear me and he hears things and then he takes out of context and maybe he doesn't understand why I feel that way or whatever. It's like I just I don't want him to hear any of like how I process my own feelings. (Participant #3)

#### The impact of stigma in help-seeking

Women experienced stigma at the personal and systemic levels which prevented some women from seeking out psychological services. In terms of barriers to receiving medical services, there was one instance where the knowledge of one perinatal woman’s career led to a few invalid assumptions of her knowledge, such as assuming that she was aware of changes that occur in the perinatal period.

##### Personal stigma

Personal stigma in terms of self-recognizing the need for professional support played an important role in initiating help-seeking behaviours. One perinatal woman shared the difficulty in acknowledging her need for support, “It was hard for me to accept it at first, so it took a long time [to go to therapy]” (Participant #5). For this participant, her social supports played an integral role in helping her eventually access professional help by providing her with the resources she needed (e.g., contact information for a counsellor). Another woman explained the challenges she experienced with personal stigma while seeking out psychological services. For this participant, the idea of needing professional support conflicted with her expectation of what health “should be”, which exacerbated her feelings of anxiety and depression in the perinatal period:

I think the reason I never really started to ask for help or anything like that was there is such a huge stigma that something was wrong with me that that you should be, I don't know why there's always expectations of what you were supposed to be and not allowing you to feel, like you shouldn't be feeling those feelings, there's something wrong with you then. (Participant #6)

##### Systemic stigma

Another emergent theme impacting perinatal women’s help-seeking behaviours was the stigma of psychological health difficulties expressed by healthcare providers. For example, prior to the perinatal period when one woman shared her experiences of anxiety and worry, her primary healthcare provider minimized her experience by saying, “anxiety is all in your head” (Participant #1). This minimization and breach of trust contributed to the experience of further psychological distress. Subsequently, when this woman experienced psychological health concerns in the perinatal period, she was unsure if she could talk to her obstetrics doctor about these symptoms due to the unsupportive interaction, she previously had with her primary healthcare provider. “I just felt like, well, that's not really his area of expertise. Like, why would I call him to make an appointment? Because of my mental health?” (Participant #1).

Additionally, one perinatal woman shared challenges with overcoming assumptions made about her level of knowledge about health during the perinatal period as a function of her occupation. This participant, who is a health care professional, felt that she was unable to express her concerns associated with her pregnancy, as her healthcare provider assumed that she knew because she was a healthcare worker. This participant described feeling dismissed and that her needs were not attended to by her health professional:

Because they knew I was a [occupation], it was just kind of like I was in and out and they didn't, or I didn't get time to ask some questions because I had those worries. I always had those worries, what if my baby's not moving? When should I? And I was kind of scared to ask them too because I thought they would judge me for being so worried or whatever, overly worried like unreasonably. And so, I kind of didn't get to answer those questions. I remember having this like lump I would feel in my belly, and I told my family doctor early, I felt that it's probably just a cyst or whatever, but to this day, nobody's actually assessed it physically. So, I kind of felt brushed off by them. (Participant #2)

### Specific Needs of Perinatal Women with a History of Interpersonal Trauma

Evident in the interviews with perinatal women, there are several specific needs relating to psychological and medical services. Three subthemes within this main theme highlight the need for a medical care team that listens and meets perinatal women’s trauma needs, medical service-related needs will be explored, as well as psychological service needs. The subtheme of medical service-related needs will be further detailed in themes including the need for trauma-informed care and “Moms need to be checked on more”. Perinatal women who had experienced trauma reflected on the need for a team of care professionals that attended to their questions directly, treated them with compassion and responded to their unique needs. Additionally, perinatal women expressed that the psychological and medical systems contain gaps from the perspectives of women with a history of interpersonal trauma and there is a need for trauma-informed care, see summary of recommendations in Table 2.

#### A medical team that listens and understands perinatal trauma needs

Emergent in the interviews with perinatal women was the various characteristics of care providers that facilitated their help-seeking experiences. These important characteristics that positively impacted their perinatal experiences included being helpful, responsive, attentive, and supportive. Many women expressed the importance of humanizing individuals in a medical system that de-humanizes. They also valued being called by their names by a healthcare provider. One woman shared the characteristics of care providers that made for a positive interaction in the perinatal period which empowered her to address her concerns:

I think just like treating us like we were people, like we mattered. And yeah, there were 10 other people in triage with me, but I never felt like my care was being cut short. I always felt like everybody was there to if we needed something, somebody was there to be able to help and stuff. So, I think it was just that feeling that like we when everybody called me by my name, it wasn't that feeling of being a number right. (Participant #1)

Throughout the interviews with women, it appeared that when care providers acknowledged women’s experiences emerged as important. When one woman spoke about experiencing difficult situations in the perinatal period, her care provider matched their emotions appropriately to the situation and acknowledged how difficult the situation must feel to the woman:

You know, like when I was in hospital and I had, like, the first time that I had blood, it was it was so much blood that it was just it was very, very concerning. And when he [Obstetrician] came to see me in the hospital the next day, he had said he's like, “I'm so sorry that this happened but baby's doing well.” You know, it was just he just always acknowledged. And I think that that was huge. It just made my experience so much better than it could have been. (Participant #7)

#### Medical service-related needs of perinatal women

Evidently, it appeared that many women’s partners were not provided with adequate information about the experiences and changes that occur in the perinatal period. Women spoke about the lack of knowledge that is also shared with partners about the types of symptoms that are a cause for concern that need to be addressed with health professionals. This was not the case for all perinatal women, as one woman shared that her partner was given information by a primary care physician about the symptoms to attend to and that warrant further attention from a health professional. This led to positive experiences for this woman as she felt supported by her partner and that he would be able to help her with her concerns if she felt that she could not address them herself.

For some women, medical appointments were especially difficult. Particularly, one woman who experienced sexual trauma prior to the perinatal period expressed that certain medical examinations were emotionally challenging and she would take prescription medication to alleviate some of her symptoms. These challenges were only exacerbated with the pandemic-related restrictions which did not allow for partners to accompany perinatal women into the exam room. One woman shared:

It was to a point where I would get a little bit of Ativan before a pap smear. And I'm not… and I don't take Ativan like all the time either, just a one-time dose before a pap because of the anxiety that I feel from having one done. But to compare my first and second pregnancies, the second time around is in COVID, and I'm not allowed to have my partner in the room with me so I'm all by myself going through all this and that. That was that was hard. There was just no support there. (Participant #8)

Trauma-informed medical care. With regards to improvements that can be made to facilitate a positive experience while seeking medical services, perinatal women spoke about the importance of a trauma-informed approach to medical care. Particularly, one woman with a history of sexual trauma prior to the perinatal period, shared that the disclosure of past trauma to medical care staff is not helpful:

I'm so sick of explaining it to providers right. I'm so sick of saying, just so you know, I was sexually abused as a child. And so, whenever you go down and you do these assessments, you need to just be really understanding of that and I'm just kind of sick of having that conversation. (Participant #8)

This woman also suggested that there are a number of changes that medical care staff can implement to create an environment that is supportive and trauma-informed. Evidently, she explained that when she went to school to become a health professional, awareness about trauma was not incorporated into learning, ultimately being a gap in the system. She also expressed after undergoing training to perform a pap smear test, she suggests that the healthcare professional should indicate initial point of contact by verbally communicating that the exam will begin followed by physical touch to the knee prior to performing the exam can be helpful when working with patients who have experienced trauma. She explained:

You know, I learned that the appropriate way to do a pap is to say the examination will now begin and say something like or I was taught to touch your hands on their knees so they feel that initial touch. And then work your way down to the area not to just go right in and just shove that speculum in right, that's what I find every doctor that I had doing an exam on me did. And not say, ‘The examination will begin.’ Nothing like that. Just boom right in. And that's every time I jumped off the bed. (Participant #8)

Similarly, one perinatal woman spoke about a challenging interaction she had when she was pregnant with a medical care provider that did not announce her actions which led to the experience of various negative emotions and left her feeling violated:

There was one nurse who, she was kind of aggressive. I don't know if I was just overly sensitive because I had been in labor for thirty-five hours. She just kind of, all the other nurses just checked you very nicely, let you know if they were going down there or anything. And she was just trying to check how dilated I was and then she just threw her glove on and literally just flipped me, shoved her hand up there and it hurt. And I had tears and I was crying because it was painful. And I remember turning to my husband, I mean, the day is a little bit of a blur now, but I remember turning to my husband being, I feel like I just got abused. (Participant #5)

Access to private rooms was a central topic discussed by many participants as in the case of pregnancy loss or complications in childbirth. Women also spoke about other challenges with sharing a room with other patients in the hospital and not having adequate privacy. One woman elaborated on the amount of distress associated with mothers sharing rooms with other mothers who were able to hold their infants:

It's hard when a mom is put into recovery with other moms, but their kid is not with them whether that is, you lost your child during childbirth, you had to go through a stillbirth, you have your kid in the NICU, it's really hard when you're in that room and you hear other moms with their crying babies around you and you can't hold yours. That's a huge thing that's really hard to deal with. (Participant #3)

*“Moms need to be checked on more.”* Women also expressed they experienced a number of challenges accessing supports. Many women expressed that there is a need for more support for those who have experienced traumatic events such as pregnancy loss and perinatal challenges such as issues with breastfeeding. A few women who had experienced pregnancy loss expressed that they experienced psychological problems but did not receive any follow-up care from trained professionals. One woman who had previously experienced pregnancy loss, expressed that there are more follow-up supports needed for women who had given birth, she explained:

I had the public health nurse call me, I think, the one-month mark just to see how I'm doing, how my feelings are. But she called again at three months to check in and because, I felt I did feel fine, I think, for the first three months. And I think that moms need to be checked on more often because we're held to such a high, I feel like people just think moms are amazing, but we're actually struggling a lot. (Participant #7)

Another perinatal woman expressed that she was experiencing psychological and physical problems associated with issues she was undergoing with breastfeeding her newborn. This woman spoke about the lack of support from trained professionals regarding challenges with breastfeeding. She expressed that when discussing breastfeeding issues with care professionals, she was told to switch to formula as opposed to identifying the reasons associated with her challenges, she explained:

Breastfeeding is hard here [in this province] because there is so little support for it. The easiest thing to do is say, “this is formula, this is safe and will help your baby right now", as opposed to having multiple trained professionals where women can seek help, that kind of thing. (Participant #9)

#### Psychological service-related needs of women

Perinatal women also made a number of suggestions that could be improved in help-seeking experiences including increasing the availability of counselling services that are easily accessible, particularly easier access to those in rural areas while also reducing the cost; training for partners that provide education on the signs and symptoms perinatal women may be experiencing; and a ‘what to expect’ guide for important areas to cover during your first psychological appointment. Women expressed how important the affordability of care is to them, and in some cases, this led to heightened distress as symptoms progressed. Had these psychological symptoms been addressed through psychological services, the prolonged experience of distress may not have occurred for these women. One woman explained that while accessing psychological services more information would be helpful in terms of what to expect at your first appointment. She elaborated:

I guess when I first started going to see a therapist, it was very unknown territory, so I didn't know what to expect. So maybe especially now, with all the technology we have and stuff you can kind of have like “what to expect at your appointment”, especially for individuals who do have anxiety. It's really hard to just go on a limb and just no problem, go to the appointment, right. I'm sure people go, get there, get in the car and decide like, “no, I'm not going in” right. (Participant #5)

This woman also spoke about how having an introductory meeting prior to the first psychological service appointment would improve her overall impression of services:

… even doing a Zoom call now. A lot of people know how to use the Internet and stuff, so maybe just doing a face to face so they can put a face to the person first and then go to the meeting. And you can kind of gauge if this is something that you would like to do or continue with or you just have a face to the person. It's really hard to just go to a meeting and spew out everything that's happened, I guess, I don't know it's really just start a conversation like that. (Participant #5)

One perinatal woman expressed that the use of more checklists would be helpful when determining if further attention is needed from a trained psychological professional. She further elaborated that when in psychological distress a symptoms checklist would be more helpful than disclosing specific details about her symptoms:

If the doctor had said, “are you experiencing ABC” or at least made it so, because I couldn't talk and I was crying nonstop when I was talking to her. So, it's easy for me to say “yes, yes, no, no, yes.” So, I think that because, when you're so sad, you don't want to go into like I'm feeling this and I'm feeling this, and I know you just want to answer yes and no. And just help me, give me something that will help me, sort of help.” (Participant #7)

Perinatal women expressed that the medical providers did not address their holistic health when providing care. A common concept that emerged in this regard was that medical providers are not asking enough questions about their psychological health, and they are also not asking these questions to their partners. Women who experienced complications in childbirth were often focused on the health and well-being of their infant, such that they neglected the distressing psychological symptoms they were experiencing. And in these cases, medical providers did not check in with the mothers with regards to their psychological health. One woman reflected on her interactions with medical providers, suggesting that further training is needed for providers to be able to ask the appropriate questions about psychological health, as it is a component of health and well-being:

I think what would be helpful is for them to ask because they think a lot of times and reasons why I never brought things up in the past is because nobody ever asked… That physical, emotional, mental and spiritual piece. That it's not just how much are you exercising what you eat, do you smoke, do you drink? Like you've asked me all the physical stuff, but you didn't ask me, how do you feel? Do you ever feel really sad? (Participant #1)

## Discussion

This study focused on understanding the experiences of nine perinatal women who experienced various types of interpersonal traumatic events and explored women’s unmet medical and psychological service needs. The grounded theory model emergent from this research described perinatal women’s experiences which consisted of five interrelated thematic categories. The main themes included: the role of trauma in women’s perinatal experiences; psychological symptoms in the perinatal period that were associated with trauma; the role of social support in women’s perinatal experiences; barriers in help-seeking experiences; and specific needs of women with a history of interpersonal trauma.

Consistent with previous research and evident in this study, interpersonal traumatic events impact women’s psychological health not only immediately following the event, but the impact of such events appeared to have lasting consequences [10, 28]. Women expressed a variety of individual traumatic events that occurred from adolescence to the perinatal period and a few women experienced more than one traumatic event. It is important to recognize that these experiences were found to lead to similar psychological symptoms of anxiety, worry, depressed mood and being overwhelmed. These symptoms coupled with the experience of distressing emotions to reminders of the traumatic event and avoiding situations that remind them of the traumatic event appear to be in line with the DSM-5 criteria for PTSS or PTSD [6]. Importantly, five participants met the threshold for probable depression based on the EPDS, four participants met the threshold for severe symptoms of anxiety according to the PASS, and three participants met the clinical cut-point for PTSD based on the PCL-5 [22–24]. Notably, women expressed that their psychological symptoms and levels of distress were exacerbated by the COVID-19 pandemic and associated visitation restrictions in hospitals and gathering restrictions in public and private residences. This finding stresses the importance of identifying and addressing emotional and mental health issues with trained professionals to mitigate the symptoms associated with the experience of trauma and its lasting impact.

The findings of this study suggest that emotional needs of perinatal women were mainly aided by means of social support. This finding is consistent with previous research indicating that perinatal women prefer to seek out emotional support from those close to them such as family and friends [17]. It is possible that women seek out informal sources of support due to the multiple barriers faced when accessing psychological services.

Consistent with previous research, interviews with perinatal women in this study found that stigma continues to impact women’s help-seeking experiences [18, 19]. Stigma was found to prevent women from addressing their psychological symptoms with a health care professional. Therefore, to help address personal stigma, providing women with appropriate self-help tools which provides links to available resources, may be helpful for women with a history of interpersonal trauma. To attend to systemic stigma, increasing awareness of such stigma among professionals and providing education on how to reduce them may be efficacious.

Findings also suggest that psychological services contain several gaps and financial barriers to access, such as cost and travelling a long distance to receive care. Services were inaccessible if they were too far away from women’s homes and too expensive while on maternity leave or while active in the workforce. Additionally, as one perinatal woman had expressed, the use of more checklists or screeners would be helpful compared to disclosing specific details to health professionals. From this perinatal woman’s perspective, it was too challenging to describe her experiences when she was in a state of psychological distress. This finding highlights the importance of effective measures to capture psychological distress which may not otherwise be shared with a clinician. Such practices may support early intervention of psychological distress. Also, increased knowledge of psychological symptoms that warrant attention from health professionals must be efficiently and effectively disseminated to perinatal women and their partners (consistent with previous research) [18]. As it currently stands, this dissemination of knowledge is not a standard practice as evidenced by the women in this study who reported that there is insufficient access to information provided by health professionals. Keeping in mind that women rely on their family and friends for emotional support, the findings of this study underscore the need for the provision of education to women’s social supports (including their partners) on issues pertaining to psychological health.

The importance of a supportive medical care staff emerged as an important component of a positive interaction with health care professionals, which was consistent with findings by Mackintosh and colleagues [29]. Findings from both studies highlighted that trust in medical care providers ameliorated some difficulties associated with past traumas. It is imperative that trauma-informed care pays particular attention to the specific qualities of the medical care staff providing care, in that the therapeutic relationship between the patient and care provider is attended to and patients are addressed by name. Evidently, medical services contain several gaps from the perspective of perinatal women who have experienced interpersonal trauma. Findings suggest several changes that can be implemented to respect women’s rights and facilitate a positive experience. It is imperative that medical professionals verbally communicate that an examination will begin to not cause distressing psychological experiences and physical violations [30]. Further, medical professionals must seek consent from women prior to performing an exam.

The findings of this study make clear that health services do not map directly on health-relevant needs of perinatal women who experienced trauma. For women who experienced pregnancy loss, many expressed that resources were not provided to them following the loss. This finding highlights a critical need to ensure that women who experience pregnancy loss are supported through health services. Additionally, many perinatal women indicated that appointments following childbirth, while focusing on the needs of the infant, should also address the needs of perinatal women. There is a need for more follow-up supports related to psychological health available to women prior to and after childbirth. Also, when continuity of care was not possible (i.e., the same doctor followed women through to the end of the perinatal period), women described a lack of comfort in disclosing their psychological health symptoms and concerns. An ongoing relationship with a known care provider was necessary and important for some women to feel comfortable thereby promoting self-disclosure, when deemed appropriate by the perinatal woman receiving care.

A few perinatal women in this study did not perceive a need to seek out professional services despite their experience of distressing psychological symptoms. In line with Cauce and associates’ (2002) help-seeking model, most perinatal women did not reach the first phase of acknowledgement and defining the problem [31]. For example, one woman described herself as a “worrier” in the context of her worrying thoughts distracting her while on the job, and at the same time describing herself as “not really a person who gets help”. This may relate to the fact that her profession is in the health care field, so she may not see herself in the ‘care receiving role’ and instead, in the ‘care providing role’. This ability to recognize perceived need for care is in part determined by psychological health literary, beliefs about health and health service use and perhaps on stigma of psychological health.

## Limitations

There are several limitations that should be considered when evaluating this study. The sample size was small and homogenous in nature, which largely consisted of educated women who were in a relationship. Additionally, given that this study represented a homogenous sample, there is a lack of a diversity with respect to sample ethnicity. As such, small and homogenous sample is a limitation with respect to the transferability of the findings of this study. Pregnant women were underrepresented in this study as most of the sample were postpartum. Additionally, since women were not recruited from hospital or clinical settings, there is a lack of representation of a clinical sample who may be experiencing different or more intense psychological impacts. Women recruited in this study may have had different experiences as compared to women recruited from a hospital setting. In the quantitative online survey, the authors asked participants to report neonatal loss but did not ask participants to report other forms of perinatal loss. Participants who may have experienced other forms of loss were not represented in this online survey. Notwithstanding these limitations, this study is the first to qualitatively explore perinatal women’s unique and diverse experiences of trauma and its subsequent impact on these women. The use of a semi-structured interview style was also a strength as this allowed for further exploration of the individual experiences of perinatal women.

## Future directions

Due to the small sample size collected in this study and the underrepresentation of pregnant women, future research should focus on hearing from a broader range of pregnant and postpartum people on their experiences with trauma and impact on medical and psychological services. Research with a nonhomogeneous sample and greater ethnic diversity may further elucidate experiences that were not captured in this study. Apparent in the findings of this study, perinatal women who experienced trauma are in need of supports relating to psychological and medical services. Findings highlight that the experience of trauma had lasting impacts on perinatal women. Additionally, the suggestions made by women in the context of medical care could improve interactions with health professionals. Importantly, the impact of context-generated trauma on perinatal women in health settings were shown to impact women beyond the perinatal period. There is a critical gap in terms of resources and supports available to perinatal women seeking psychological and medical care from a trauma-informed lens. Such programs may help alleviate the lasting impact of trauma and related psychological symptoms.

## Conclusions

According to the World Health Organization, poor psychological health during the perinatal period is a significant public health issue [32]. However, research on interpersonal trauma does not reflect the magnitude of concern for these experiences and their impact on perinatal women. The current study was novel in its exploration of interpersonal trauma and its impact on perinatal women, perinatal experiences during the COVID-19 pandemic, social support and its value when present, barriers experienced by perinatal women while seeking medical and psychological services, and unmet health needs which points to the need for care that attends to the all-inclusive and trauma-informed needs of perinatal women. To summarize the quantitative findings, considering that several women met the threshold for depression, anxiety and/or PTSD, and a number of which did not seek out professional care, this highlights the need for increased services and programs that promote mental health literacy in this population. To this end, this research is especially important in elucidating the nuances of perinatal women’s experiences the context of perinatal care which are be important for service providers involved in the psychological and medical care of perinatal women. Further, the findings of this study inform future supports and resources for perinatal women.

## Supplementary Information

**Additional file 1: Appendix A.** Semi-Structured Qualitative Interview Protocol.

**Additional file 2: Appendix B.** Thematic Framework with Quotations from Participants.

## Data Availability

The transcripts used and analyzed during the current study are available from the corresponding author on reasonable request.

## Abbreviations

PTSS: Posttraumatic stress symptoms
PTSD: Posttraumatic stress disorder
EPDS: Edinburgh Perinatal/Postnatal Depression Scale
PASS: Perinatal Anxiety Screening Scale
PCL-5: The Posttraumatic Stress Disorder Checklist for the DSM-5
