# Supplementary material for: Women’s experiences of trauma, the psychosocial impact and health service needs during the perinatal period
Source: BMC Pregnancy Childbirth. 2023 Mar 21;23:197. doi: 10.1186/s12884-023-05509-5 (PMC10028773; doi:10.1186/s12884-023-05509-5)
Supplement: Supplementary file 1 — Additional file 1: Appendix A. Semi-Structured Qualitative Interview Protocol. [file 12884_2023_5509_MOESM1_ESM.docx]

Appendix A

Semi-Structured Qualitative Interview Protocol

Thank you for coming in today. As you know, the purpose of this research is to gain a better understanding of the ways in which traumatic events and posttraumatic stress symptoms impact women’s experiences in pregnancy and postpartum, including their healthcare service use (mental and physical). In the interview, we will ask you questions about your mental health, perinatal health, and healthcare service use and experiences with services. Please answer as freely as possible. If you do not want to answer certain questions, please let us know and we will move on to the next questions. If you feel yourself getting upset or needing a break, we can take a break or stop the interview.

A. Has there ever been a time in your life where you have experienced difficulty with an emotional or mental health problem relating to a traumatic experience (distress, anxiety, depression, trauma)?

1. If you are comfortable, would you be able to tell me about the experience in a sentence or two? I don’t need a lot of detail, just enough to understand what happened.

2. How do you think this experience has impacted your life?

o How has it impacted your experiences during pregnancy?

o (If postpartum) After giving birth?

o (If postpartum) How has it impacted your experiences in adjusting as a mother?

B. Did you receive any professional help/support relating to this experience?

1. Were there any barriers to you receiving/accessing professional help/support?

o If YES: a) In what way(s) do you think that barrier stopped you from seeking help? How would you like to see that barrier removed or changed? b) Was the support you received helpful for you?

• (If yes) What made it helpful?

• (If no) What do you think would have made it more helpful to you?

• If NO If you were to seek help, what would you like services to look like? What would have bene helpful to you?

C. What was it like for you to pursue medical appointments during the pregnant and or postpartum period?

1. How did the traumatic experiences that you experienced impact this?

2. What about your interaction with healthcare providers?

3. Is there anything that would have helped with this? Probe for: Services? Medical protocols? Staff approaches?

D. How would you describe your emotional or mental health now?

E. What has contributed to the way that you are feeling now? What has been helpful in improving mental health? What has contributed to worsening mental health?

F. Is there anything else that you would like to say before we end the interview?
