# Supplementary material for: Women’s experiences of trauma, the psychosocial impact and health service needs during the perinatal period
Source: BMC Pregnancy Childbirth. 2023 Mar 21;23:197. doi: 10.1186/s12884-023-05509-5 (PMC10028773; doi:10.1186/s12884-023-05509-5)
Supplement: Supplementary file 2 — Additional file 2: Appendix B. Thematic Framework with Quotations from Participants. [file 12884_2023_5509_MOESM2_ESM.docx]

Appendix B

Thematic Framework with Quotations from Participants

| Main Theme | Subtheme | Participant Exemplar Quotes |
| --- | --- | --- |
| The Role of Traumatic Events in Women's Perinatal Experiences | Impact of traumatic events on daily perinatal life | • Even right now, it's seeing scenes of childbirth, like in movies and TV shows, I will do something else when that is playing on the TV, I will completely distract myself and do the dishes and play with my [baby] and do a whole bunch of stuff so I'm not paying attention to that scene. Because I want to cry or scream or just shout, why didn't I get that [childbirth experience] I didn't get that at all. (Participant #3)  • I had never had to... be worried about like living or dying before, I never had that concept before [traumatic event] so now it just makes you not like appreciate things more, but just to be more aware of the seriousness of certain things. (Participant #4)  • The postpartum period was a huge adjustment for me. I know it’s like that for lots of women I guess, but I don’t know if it was worse because of my previous [traumatic] experiences. But I did have the postpartum blues. It went away after two or three weeks; I was still moody but not crying. But there were a few days postpartum where I would just cry uncontrollably. (Participant #2)  • We lost three babies before my [daughter] and the anxiety of, just even the fear of losing her was there consistently. And the fear that something was always going to be wrong was always there. And it was really hard to enjoy being pregnant because it was so fearful. (Participant #6)  • I was on sick leave for the last couple of months of my pregnancy and I definitely reverted back to staying in bed all day. I tried my hardest not to not to feel the feelings… I did a lot of journaling, which is something that I have never faithfully done. I would start in the past with good intentions and then it would kind of just go to the wayside. But it was really, in my mind, the only thing I can really do. (Participant #8) |
|  | Psychosocial impact of traumatic events | • I felt a lot of, very overwhelming, the fact that I couldn't help her [baby] in any way, I felt completely helpless and a lot of that definitely felt very familiar too. I felt very helpless in processing everything that I had to do with my divorce. I couldn't, it was happening, and I felt like I lost complete control of my life. And when my [baby] was born and she wasn't with me, I felt like I again lost complete control of everything. (Participant #3)  • “overwhelming sadness” {for many years following traumatic event} [The traumatic event] was in [year]. It probably took me, I would say, when I had my [baby] six months ago [six years later] to kind of recover from that. (Participant #7)  • {Following traumatic event} There was just this feeling of like I can’t do this, like I don’t want all those overwhelming feelings.... It’s more of this like I can’t breathe, I feel super nauseous, I can’t eat anything. I just don’t want to get out of bed, like all of those kinds of depression, anxiety, feelings. (Participant #1)  • I just was very stressed out and just didn’t want to deal with anything anymore (Participant #5)  • It was very traumatic mentally, and I struggled with a lot of depression after. And since then, I think, I got postpartum depression and anxiety after being pregnant because of that. (Participant #6) |
|  | Pressure to make right decisions for pregnancy and for baby | • Her [baby] well-being was only fully in my hands because I was literally the only source of food for her. (Participant #1).  • There's a lot of unknown and with that comes a lot of fear and anxiety of trying to be the right parent, even though everybody has so many different styles of how to parent. And just trying to like, there's so much new information out there that how do you know what's the right thing to do? (Participant #3)  • While also not sleeping because I had a newborn and dealing with my own pain [with breastfeeding] and trying to sort out breastfeeding issues and trying to get help with that. Yeah, it was it was very, very hard. (Participant #9)  • I felt like I myself, I was so diligent. It was my first pregnancy. I did everything that you're supposed to do to have a healthy pregnancy. I went to all my prenatal visits… But I just felt like how could this, what did I do in my life as a caring [healthcare provider] to deserve something like this [traumatic event] to happen to me? (Participant #7) |
| Perinatal Experiences in the COVID-19 Pandemic | Impact of COVID-19 on the perinatal period | • “highs and lows” (Participant #5)  • I think [the COVID-19 pandemic] exacerbated every symptom. Because we're not supposed to just live in our houses and be isolated. And I'm very, very social, and very extroverted. So, we literally went from like when I was off with my [baby], we literally went from being in run time, and a ‘mom and me’ group and going to like a feeding support group and going to the going to swimming lessons. We had something every single day of the week to nothing…And so I found myself talking to the dog and I just felt like just I guess just not having the supports there. (Participant #1)  • I think the pandemic actually kind of had as crazy it sounds a positive twist because I don't know if you know, but like the hospitals, when the pandemic first hit, people were scared to go. My job went from being so busy and stressful to, like, relaxed I could spend time with my patients. (Participant #2)  • So there's definitely days where I just I feel like a moping around sometimes. Yeah you just can't invite anyone over because of COVID, you can't do anything so you just feel like at wits end a little bit. But at the end of the day, I just always remember, like, I get to be at home safe and I have this cute freaking kid at home (Participant #5)  • Not having a support person coming in with you [at hospital appointments] to support you, because you have dealt with so much trauma, was horrible. And then going in multiple times was even harder because you’re just waiting for bad news to show up. (Participant #6)  • COVID is unbelievable. I don't even know it's so hard to just to have those sleepless nights and take care of my four-year-old with a newborn when you're completely exhausted. Well, I think if COVID wasn't around and I could just, you know, take my daughter to [kids gym] and just let her play while I just sit and, you know, nurse or do whatever to my baby, then that would be ideal. I think that that would make such a huge difference but can't do those things. I do feel like I do wonder, if I wasn't if it wasn't a pandemic right now and I could use the resources that like my in-laws or the swimming or whatever with my daughter, I do wonder if I would still have been diagnosed with depression and I do wonder that. (Participant #7) |
|  | Altered expectations of the perinatal period due to COVID-19 | • Missing out… I'm also slightly disappointed because my expectations for my maternity leave are very, very different than what my expectations have been. And then to maybe like a little bit feeling of guilt just for my [baby], the social aspect so, I know it has nothing to do with me necessarily, but just feeling a little guilty that she might be missing out on all these great plans that I had for the first year. (Participant #4)  • Had worries about getting COVID being at the hospital giving birth, worried that anyone who comes near baby has COVID or baby will get COVID. And didn’t get to have people come over and help support her with baby. (Participant #5)  • There were so many things I wanted to do, things that I looked forward to having, like baby showers and just being able to get together with friends and their kids. All the things you look forward to for so long and then being taken away from you because you can’t get together… you feel like there’s just no one there because it’s so isolating at times. (Participant #6)  • But to compare my first and second pregnancies, the second time around was in COVID and I'm not allowed to have my partner in the room with me so I'm all by myself going through all this and that. That was that was hard. There was just no support there. (Participant #8)  • We didn't have a shower, and we didn't have lots of things that you might have had otherwise [if it weren’t for the pandemic]. (Participant #9) |
| The Role of Social Support in Perinatal Experiences | The "healing" impact of social support | • [Husband would] Just being an empathetic, listener, instead of just saying, "oh, no, you're fine, you're fine like, cheer up.” I think it would be a lot more difficult [without the support]. I think it stopped any of those [negative] feelings from becoming prolonged. I think it helped me deal with it, not quicker, but just to heal faster. So, I think that was good. (Participant #4)  • Talking openly with friends was pretty like, I don’t want to say liberating, it was just sort of like a weight off my chest. (Participant #1)  • I just have a very open relationship with the both of them [husband and mother-in-law] so I feel like I can speak whatever I’m feeling with them and it’s no judgement… I can just say something and she completely 100% understands. (Participant #5)  • For the most part, he [husband] will make sure that I am getting together with someone or talking with a friend or giving me just even the evening off to go grocery shopping to get away from all the stress at home and to kind of allow me to decompress my day. (Participant #6) |
|  | Importance of validating perinatal experiences | • A car always needs to be [maintained], right. Like, you got to go for an oil change. So, you're essentially going for your own personal oil change. (Participant #5).  • It was more beneficial than for me going to a counselor because the counselor didn't really know how to feel those feelings or to know what it's like to lose a child or anything like that. Then once I found a group of friends that did, it was more benefit because I could feel like, ok, I know that these are just feelings and I'm allowed to feel them and they're normal because they're feeling them too. So, it just made me feel a lot more normal as a human being to have a group of people that I can talk to. (Participant #6)  • Talking with other friends who have had babies helped. I had opened up to one of my really close friends who has two little girls under four and shared some of the struggles with her. And she’s like “I felt all of that”. And I was like, but when I didn’t have kids and I asked you how things were going you said everything was great. And she’s like, “I don’t know, because we’re always just told that motherhood is supposed to be this beautiful time. And you don’t share your struggles because you should be grateful that you have a baby. And then when I start talking to other people and saying that I am having a difficult time, I found that there were a lot of women who had similar, if not worse experiences that I had, just nobody was talking about it. (Participant #1). |
|  | Challenges of a limited social network | • You feel just there's no one there because it's so isolating at times, especially because my husband is only home for two hours before the kids go to bed, so I have them for the majority of the time and my [baby] is only one here, and so she's not really talking so I don't have anyone to talk to all day except for two screaming children that can't tell me what they want. (Participant #6)  • Lack of social support from mom “the first time I told her that I had depression, she said, ‘don't just assume you have that’ So then right away that's like a negative experience.” (Participant #5) |
| Barriers in Help-Seeking Experiences | Practical barriers | • I found that I fell into this gap where I had no benefits while I was on maternity leave and I was on employment insurance, so paying a hundred and fifty dollars for a psychologist each time wasn't something that was realistic for us. (Participant #1)  • No one's taking new clients, limited hours, limited services. (Participant #8)  • Talking about it at home, it's like I don't know if my boyfriend can hear me and he hears things and then he takes out of context and maybe he doesn't understand why I feel that way or whatever. It's like I just I don't want him to hear any of like how I process my own feelings. (Participant #3)  • It’s [therapist appointments] just expensive. After university, I saw my therapist but it’s in the city, and now I live [out of the city] so it’s far and it’s a really big commitment to always go. (Participant #5)  • I think that in a different time [not in COVID], I would have been around a lot more people in my prenatal time and probably would have had more conversations with moms and then maybe been pointed toward taking a breastfeeding class. Participant #9) |
|  | Impact of stigma in help-seeking | Personal stigma  • It was hard for me to accept it at first, so it took a long time [to go to therapy] (Participant #5)  • I think the reason I never really started to ask for help or anything like that was there is such a huge stigma that something was wrong with me that that you should be, I don't know why there's always expectations of what you were supposed to be and not allowing you to feel, like you shouldn't be feeling those feelings, there's something wrong with you then. (Participant #6)  • There wasn’t a lot of resources I could get into, and when I was [younger] I was like ‘I don’t need this, I don’t have a problem’ So I think it was just hard to kind of acknowledge that or have resources to help me understand that (Participant #1)  • I kind of just wanted to wait it out. My mom warned me about how you may have postpartum blues, but I didn’t think I would need it [help]. I kind of just view myself as somebody who wouldn’t need professional help or anything. (Participant #2)  Systemic stigma  • {Doctor had said} “anxiety is all in your head”. (Participant #1)  • Because they knew I was a [occupation], it was just kind of like I was in and out and they didn't, or I didn't get time to ask some questions because I had those worries. I always had those worries, what if my baby's not moving? When should I? And I was kind of scared to ask them too because I thought they would judge me for being so worried or whatever, overly worried like unreasonably. And so, I kind of didn't get to answer those questions. I remember having this like lump I would feel in my belly, and I told my family doctor early, I felt that it's probably just a cyst or whatever, but to this day, nobody's actually assessed it physically. So, I kind of felt brushed off by them. (Participant #2)  • {Doctor brushed off concerns related to measurements and expected growth during pregnancy} “you’re a fit girl so you won’t grow like a lot of other women” (Participant #7)  • I found that I would tell my doctors about this [worries] but there wasn’t really anyone who was like ‘oh yeah here let me listen to what you have to say’ they would just kind of push me away and just say “well you can go see this person” instead of just actually listening to my fears and concerns. (Participant #6) |
| Specific Needs of Women with a History of Trauma | A medical team that listens and understands perinatal trauma needs | • I think just like treating us like we were people, like we mattered. And yeah, there were 10 other people in triage with me, but I never felt like my care was being cut short. I always felt like everybody was there to if we needed something, somebody was there to be able to help and stuff. So, I think it was just that feeling that like we when everybody called me by my name, it wasn't that feeling of being a number right. (Participant #1)  • You know, like when I was in hospital and I had, like, the first time that I had blood, it was it was so much blood that it was just it was very, very concerning. And when he [Obstetrician] came to see me in the hospital the next day, he had said he's like, “I'm so sorry that this happened but baby's doing well.” You know, it was just he just always acknowledged. And I think that that was huge. It just made my experience so much better than it could have been. (Participant #7)  • I found that the public health nurse visit I had postpartum was very helpful. I found it very helpful to have a face-to-face. And they kind of just reassured me. They didn’t make any questions feel dumb and they gave me lots of pamphlets… It was very helpful at that time (Participant #2)  • The help that I’ve had, especially around my pregnancy, the more sensitive the nurses are to each mom’s situation, the better. (Participant #3)  • Just by addressing individuals by their name and remembering that type of stuff that’s what I really have valued, like when nurses or clinicians remember your name it just helps. And if they just remember things that you’ve talked about, and I know they’re not superhuman but it’s just nice if they review your file right before. It’s nice when they remember little things because then you don’t have to repeat yourself constantly because it’s very daunting when you have to repeat your story over and over, especially if you’re meeting with the same people all the time. (Participant #5) |
|  | Medical service-related needs | Trauma-informed care  • It was to a point where I would get a little bit of Ativan before a pap smear. And I'm not… I don't take Ativan like all the time either, just a one-time dose before a pap because of the anxiety that I feel from having one done. But to compare my first and second pregnancies, the second time around is in COVID, and I'm not allowed to have my partner in the room with me so I'm all by myself going through all this and that. That was that was hard. There was just no support there. (Participant #8)  • I'm so sick of explaining it to providers right. I'm so sick of saying, just so you know, I was sexually abused as a child. And so, whenever you go down and you do these assessments, you need to just be really understanding of that and I'm just kind of sick of having that conversation. (Participant #8)  • You know, I learned that the appropriate way to do a pap is to say the examination will now begin and say something like or I was taught to touch your hands on their knees so they feel that initial touch. And then work your way down to the area not to just go right in and just shove that speculum in right, that's what I find every doctor that I had doing an exam on me did. And not say, ‘The examination will begin.’ Nothing like that. Just boom right in. And that's every time I jumped off the bed. (Participant #8)  • There was one nurse who, she was kind of aggressive. I don't know if I was just overly sensitive because I had been in labor for thirty-five hours. She just kind of, all the other nurses just checked you very nicely, let you know if they were going down there or anything. And she was just trying to check how dilated I was and then she just threw her glove on and literally just flipped me, shoved her hand up there and it hurt. And I had tears and I was crying because it was painful. And I remember turning to my husband, I mean, the day is a little bit of a blur now, but I remember turning to my husband being, I feel like I just got abused. (Participant #5)  • It's hard when a mom is put into recovery with other moms, but their kid is not with them whether that is, you lost your child during childbirth, you had to go through a stillbirth, you have your kid in the NICU, it's really hard when you're in that room and you hear other moms with their crying babies around you and you can't hold yours. That's a huge thing that's really hard to deal with. (Participant #3)  "Moms need to be checked on more" (Participant #3)  • I had the public health nurse call me, I think, the one-month mark just to see how I'm doing, how my feelings are. But she called again at three months to check in and because, I felt I did feel fine, I think, for the first three months. And I think that moms need to be checked on more often because we're held to such a high, I feel like people just think moms are amazing, but we're actually struggling a lot. (Participant #7)  • Breastfeeding is hard here [in this province] because there is so little support for it. The easiest thing to do is say, “this is formula, this is safe and will help your baby right now", as opposed to having multiple trained professionals where women can seek help, that kind of thing. (Participant #9)  • I know that my other friends that have been pregnant were screened for everything like health, mental health. But no one did that for me. I think if they had done that and knew that like “Oh she can be a bit anxious and like a worrywart” maybe they would have gone into that further in any of my visits. But nobody did any screening questions. (Participant #2)  • I don’t think I really realized that I was dealing with a lot of stuff with my mental health while I was in the hospital. I was so focused on my daughter’s health being ok, and dealing with the recovery of my surgery from the C-section and my physical body just trying to recover that way that I really didn’t notice that I was dealing with so much on my mental health. (Participant #3)  • There is no group around that I know of in [province] that deals with stuff like pregnancy loss, that allows people to get together with each other like a support system. I think that having something like that would be so important and having someone that has professional education and how to deal with a group. (Participant #6) |
|  | Psychological service-related needs | • I guess when I first started going to see a therapist, it was very unknown territory, so I didn't know what to expect. So maybe especially now, with all the technology we have and stuff you can kind of have like “what to expect at your appointment”, especially for individuals who do have anxiety. It's really hard to just go on a limb and just no problem, go to the appointment, right. I'm sure people go, get there, get in the car and decide like, “no, I'm not going in” right. (Participant #5)  • … even doing a Zoom call now. A lot of people know how to use the Internet and stuff, so maybe just doing a face to face so they can put a face to the person first and then go to the meeting. And you can kind of gauge if this is something that you would like to do or continue with or you just have a face to the person. It's really hard to just go to a meeting and spew out everything that's happened, I guess, I don't know it's really just start a conversation like that. (Participant #5)  • If the doctor had said, “are you experiencing ABC” or at least made it so, because I couldn't talk and I was crying nonstop when I was talking to her. So, it's easy for me to say “yes, yes, no, no, yes.” So, I think that because, when you're so sad, you don't want to go into like I'm feeling this and I'm feeling this, and I know you just want to answer yes and no. And just help me, give me something that will help me, sort of help.” (Participant #7)  • I think what would be helpful is for them to ask because they think a lot of times and reasons why I never brought things up in the past is because nobody ever asked… That physical, emotional, mental and spiritual piece. That it's not just how much are you exercising what you eat, do you smoke, do you drink? Like you've asked me all the physical stuff, but you didn't ask me, how do you feel? Do you ever feel really sad? (Participant #1)  • The more available counselling is, the better. (Participant #3) |
